# Effect of the COVID-19 pandemic on colorectal cancer screening in two university-affiliated health care systems

**DOI:** 10.1371/journal.pone.0317057

**Published:** 2025-01-06

**Authors:** Vinod Kumar, Lilian Golzarri-Arroyo, Sarah Roth, Thomas F. Imperiale

**Affiliations:** 1 Division of General Internal Medicine and Geriatrics, Indiana University, Indianapolis, IN, United States of America; 2 Department of Epidemiology and Biostatistics, Indiana University School of Public Health-Bloomington, Bloomington, IN, United States of America; 3 Center for Health Services Research, Regenstrief Institute Inc, Indianapolis, IN, United States of America; 4 Division of Gastroenterology & Hepatology, Department of Medicine Indiana University School of Medicine, Indianapolis, IN, United States of America; Washington University in St Louis, UNITED STATES OF AMERICA

## Abstract

**Objectives:**

In two large university affiliated healthcare systems, we examined trends in colorectal cancer (CRC) screening both prior to and during the COVID-19 pandemic to compare the trends in non-invasive screening tests and colonoscopy.

**Materials and methods:**

In this retrospective time-trend analysis, we obtained the numbers of colonoscopies and non-invasive tests performed monthly during the pandemic and the year prior to it. We obtained colonoscopy data from five endoscopy units with the indication determined by dual independent review. Monthly numbers of completed fecal immunochemical (FIT) and FIT-DNA tests were obtained from the electronic medical records of both health systems. Trends in testing, numbers, and stage of incident CRCs diagnosed during the 30-month interval were examined using Poisson regression and logistic regression, respectively.

**Results:**

From January 2019 to June 2021, we identified 16,939 FIT tests, 2,942 FIT-DNA tests, and 38,332 colonoscopies from the two health systems, and 368 colorectal cancers (105 early stage, 263 advanced stage) from the private hospital system. Overall colonoscopy volume declined by 18.7% (from 16,483 to 13,393) in 2020 compared to 2019 in both health systems, returning to baseline in 2021. Non-invasive tests declined by 21.9% in 2020, but increased in 2021 due to greater use of FIT/DNA. Compared to 2019, incident CRCs declined in 2020 but rebounded in 2021, with no difference in early versus late-stage cancers.

**Conclusions:**

These trends in CRC screening tests may be useful for modeling the effects of the pandemic on the longer-term outcomes of CRC incidence and mortality.

## Introduction

Colorectal cancer (CRC) is the second most common visceral cancer in the U.S. in men and women combined and the second-leading cause of cancer mortality [1, 2]. Screening for CRC reduces both the incidence of and mortality from CRC and is cost-effective [3, 4]. Recent data indicate that roughly 72% of persons ages 50–75 years are up-to-date with CRC screening [5]. Among the more recently recommended 45–49-year-old age group, it is too early to know, with reasonable certainty, what percent is current with screening [6, 7].

The COVID-19 pandemic resulted in dramatic upheaval and rapid transformation within all sectors of the health care system, including emergent, urgent, and elective admissions and procedures, patient-provider encounters, and provision of preventive services [8, 9]. The effect of the pandemic on all sectors of healthcare raised concern for an anticipated decline in health and health outcomes in the population. CRC is one cancer expected to increase because of the shelter-in-place orders during the spring of 2020, during which time there was a precipitous decline in both screening and diagnostic colonoscopy for positive non-invasive tests. One modeling study based on early pandemic declines in screening estimated 10,000 additional deaths from CRC and breast cancer in the U.S. [8].

Several studies reported declines in CRC detection and colonoscopy volumes early during the pandemic. A U.S. study from a large integrated health care organization reported cessation of mailing of fecal immunochemical (FIT) kits in March and April of 2020, followed by a nearly 9% increase in kits mailed in 2020 compared with 2019 [10]. This study also reported a 27% decline in colonoscopy volume in 2020 compared with 2019, along with a 27% decline in CRC diagnosis during the same 2-year period [10]. None of these studies examined screening test trends and CRC diagnoses beyond December 2020 [10, 11].

With the limitations of previously reported data in mind, the primary objective of this study was to examine and compare trends in non-invasive screening tests and colonoscopy both during and prior to the pandemic within two large, university-affiliated, integrated healthcare systems within Marion County, IN, examining trends beyond 2020. A secondary objective was to examine the number and stage distribution of CRCs within the larger of the two systems over the same period. These data may be useful for estimating the effects of the pandemic on the future effects of CRC incidence and stage distribution within Marion County.

## Materials and methods

### Study design

This study is a retrospective time-trend analysis of screening tests (both invasive and non-invasive) for CRC in central Indiana before and during the COVID-19 pandemic, extending from January 2019 through June 2021. This study was conducted in accordance with the Declaration of Helsinki. The Institutional Review Board (IRB) of Indiana University acted as the central IRB, whose review was accepted by all participating institutions’ IRBs. The central IRB determined that this research involved minimal risk and approved a waiver for informed consent (Protocol #2009884510) due to the minimal risk nature of the study and aggregate nature of the data. All methods were carried out in accordance with Indiana University regulations and guidelines.

### Study setting

Data were gathered from two large integrated university-affiliated health systems located in central Indiana and comprised of a private hospital system present throughout Indiana (with the current analysis limited to facilities in Marion County) and a public hospital system with a network of Federally Qualified Health Centers. We compared the numbers of noninvasive and invasive screening tests performed monthly during time of the pandemic (4/1/2020 through 6/30/2021) and a pre-pandemic historical time frame (1/1/2019-3/31/2020).

### Data sources

For colonoscopy, personnel at five endoscopy units (four from the private hospital system and one from the public hospital system) provided numbers of outpatient colonoscopies performed per month from January 2019 through June 2021. Procedure-level data, including indication, were available from three of the five centers. Colonoscopies with an indication of patient participation in a clinical trial were excluded. Remaining listed indications were categorized as screening, diagnostic (including those done for a positive FIT or FIT-DNA test), surveillance, or therapeutic (large polyp removal, stent insertion, etc.) by consensus of two authors. Colonoscopies with listed indications in more than one category were categorized by ranking surveillance over screening, followed by diagnostic, and lastly therapeutic. In cases where a patient had more than one colonoscopy with the same indication within 7 days, only the latest one was counted. Colonoscopies performed within 7 days with different indications were categorized and included or excluded by consensus of the authors. Colonoscopies via stoma or endoscopy of a Hartman pouch were excluded.

For non-invasive tests, we collected the total number of stool-based tests (FIT and FIT-DNA) completed per month from outpatient clinics in the same geographic area through either hospital system between 1/1/2019 and 6/30/2021. Monthly numbers of completed FIT and FIT-DNA tests were obtained from the electronic medical records of both health systems and aggregated by data services at the Regenstrief Institute, Inc. While FIT was well-established in both hospital systems prior to the pandemic, FIT-DNA had been sparsely used in the private hospital system prior to the pandemic and was not available in the public system at any time.

The total number of newly diagnosed CRCs, with staging based on the American Joint Committee on Cancer (AJCC) staging system, was obtained from January 2019 through November 2021 from the private hospital system’s pathology database. We then reviewed the electronic medical record of those patients to ascertain disease stage.

### Data management

De-identified data were accessed via medical records on June 30, 2022. Relevant data were transmitted securely to the research team as Microsoft Excel spreadsheets and stored in a secure folder accessible only by the authors. Spreadsheets for each month and endoscopy unit were combined into a single spreadsheet for non-invasive tests and one for colonoscopies, insuring consistent formatting. Descriptive statistics, tables, and figures were produced in Microsoft Excel.

### Analysis

Data were analyzed using a Poisson regression to compare the number of monthly colonoscopies and FIT/FIT-DNA tests by year from 2019 to 2021. Poisson regression was also used to compare the number of monthly screening tests by health system by year from 2019 to 2021. Linear regression was performed to compare the monthly proportion of non-invasive screenings by health system over time. Logistic regression was performed to compare the odds ratio of various stages of CRC as early-stage cancer (AJCC stages I & II) and advanced stage cancer (AJCC stages III & IV).

## Results and discussion

A total of 16,939 FIT tests, 2,942 FIT-DNA tests, and 38,332 colonoscopies were performed during the 30-month study period from the two health systems combined. A total of 368 colorectal cancers (105 early stage, 263 advanced stage) were identified from January 2019-December 2021 within the private hospital system.

### Colonoscopy

**Fig 1:**
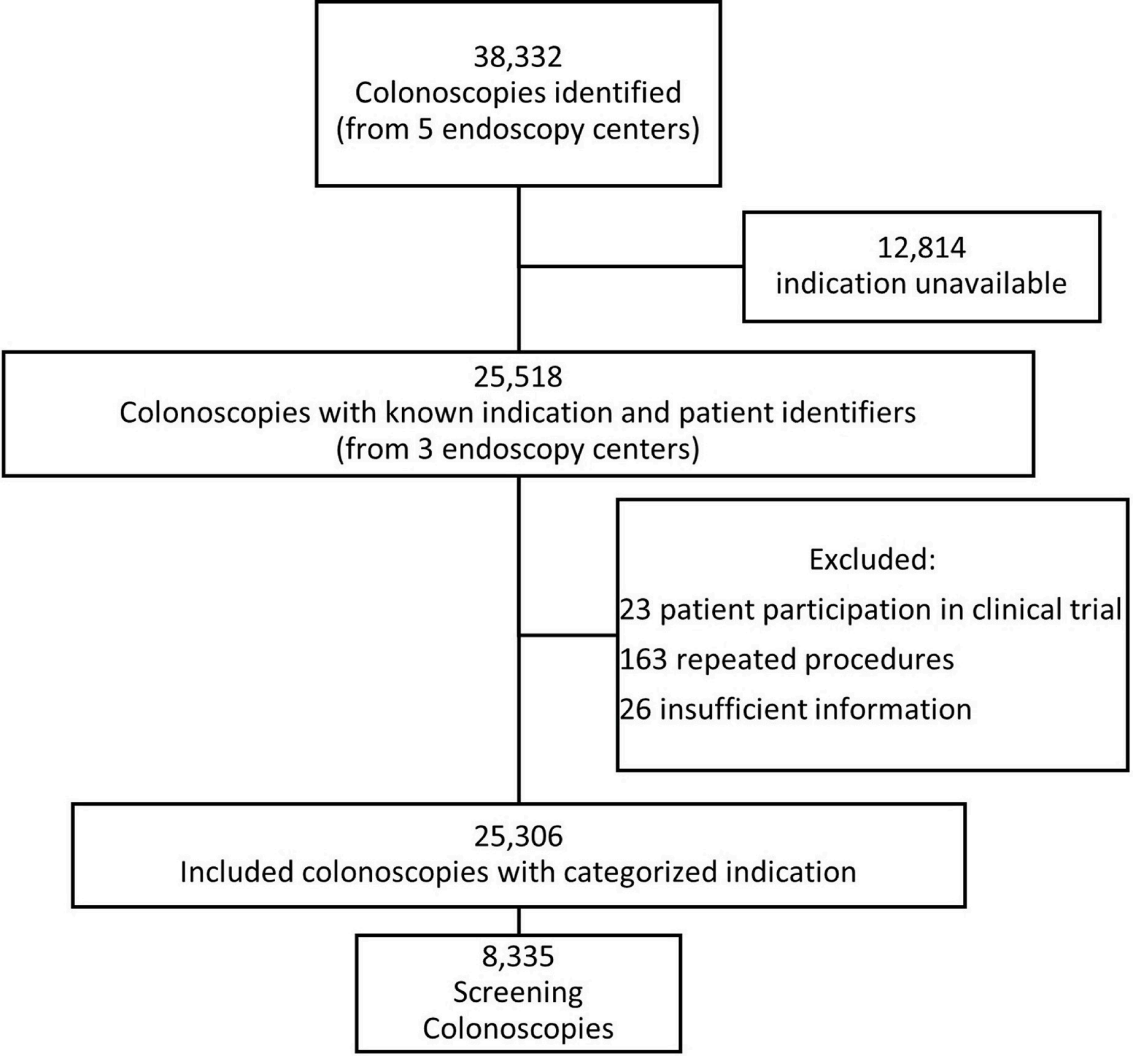
Categorization of completed colonoscopies.

A total of 38,332 colonoscopies were performed during the study period of 01/01/2019 to 06/30/2021 from the two health systems combined, with 30,775 performed in the private health system and 7,557 in the public health system. Procedure-level data, including indication, were available for 25,518 (66%) colonoscopies from three endoscopy centers. We excluded 23 colonoscopies for patients who were in clinical trials, and 163 for those who had repeat procedures within 7 days with the same indication. We further excluded 26 procedures where the recorded indications were insufficient for categorization, leaving 25,306 for final analysis. (**Fig 1**). Of the included colonoscopies with recorded indication, 8,335 (32.9%) were categorized as screening, of which the private health system had 5,287 and the public health system had 3,048 screening colonoscopies.

Overall colonoscopy volume declined by 18.7% (from 16,483 to 13,393) in 2020 compared to 2019 in both health systems. There was 17.2% decline from 13,169 to 10,903 in the private hospital, and a 24.9% decrease from 3,314 to 2,490 in the public hospital, indicating a significantly larger decrease for the public hospital system (p = 0.001). The major decline in the number of colonoscopies occurred from April 2020 through August 2020, where overall volume declined by 35.3% (from 5,384 to 3,481) in the private health system and a significantly larger decrease of 43.2% (from 1,411 to 802) in the public health system (p = 0.010), as compared with the same five-month period in 2019 (**Table 1**).

**Table 1:** Colonoscopy with known indication by year.

	Surveillance	Screening	Diagnostic	Therapeutic	Total
**2019**	4176	3715	2246	359	10496
**2020**	3944	2723	2083	296	9046
2021*	2308	1897	1372	187	5764
**Grand Total**	**10428**	**8335**	**5701**	**842**	**25306**

* First six months of 2021

**Fig 2:**
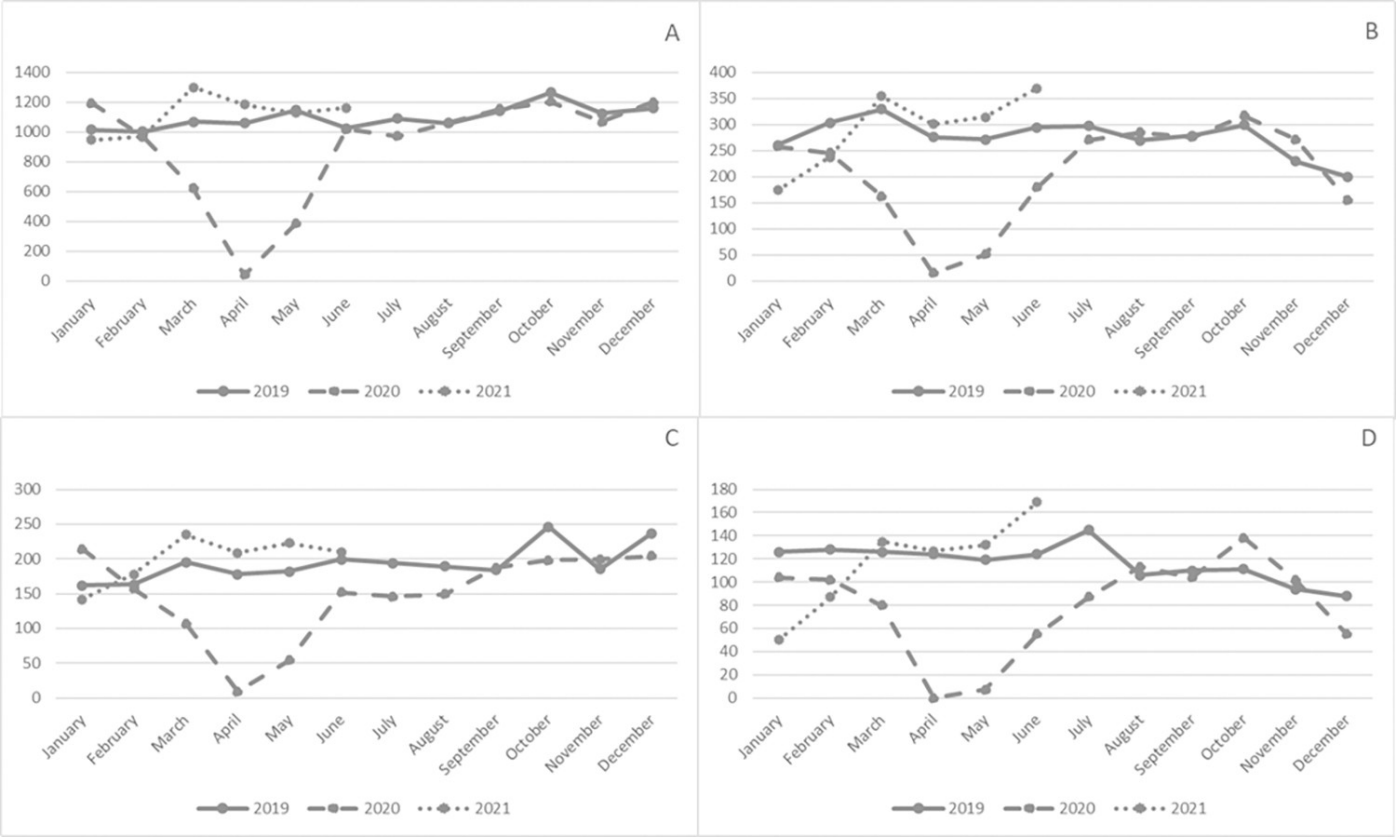
Colonoscopies performed per month at two health systems. **A**. Total colonoscopies performed through the private hospital system. **B**. Total colonoscopies performed at the public hospital system. **C**. Screening colonoscopies performed at the private hospital system. **D**. Screening colonoscopies performed at the public hospital system.

Compared to 2019, the screening colonoscopy volume in both systems combined declined by 26.7% (from 3,715 to 2,723) in 2020. Month-by-month comparisons show that screening colonoscopies for both health care systems decreased dramatically in April of 2020, but returned to pre-pandemic levels by early fall of 2020 (**Fig 2**). Compared to April through August 2019, screening colonoscopies from April 2020 through August 2020 declined by 45.9% (from 942 to 510) in the private hospital system and by 57.6% (from 618 to 262) in the public hospital system, a significantly larger decline for the public hospital system (p = 0.009). Screening colonoscopies performed between April 2020 and December 2020 declined by 27.6% in the private hospital and by 35.3% in the public hospital compared to the same months in 2019.

Screening colonoscopy volume declined more in the public hospital system compared to the private health system, with a marginally statistically significant difference in the extent of decline between two health care systems (p = 0.077). Diagnostic colonoscopy volume decreased 7.3% (from 2246 to 2083) between 2019 and 2020, and surveillance colonoscopy volume declined 5.6% (from 4176 to 3944), with no significant difference in diagnostic colonoscopy volume between systems (p = 0.64). During January through June 2021, the total number of colonoscopies increased 64.1% (from 5,152 to 8,456) and screening colonoscopies increased 82.2% (from 1,041 to 1,897) compared to January through June 2020, and were similar to 2019 January through June numbers: 8,062 total colonoscopies and 1,826 screening colonoscopies.

### Non-invasive tests

**Fig 3:**
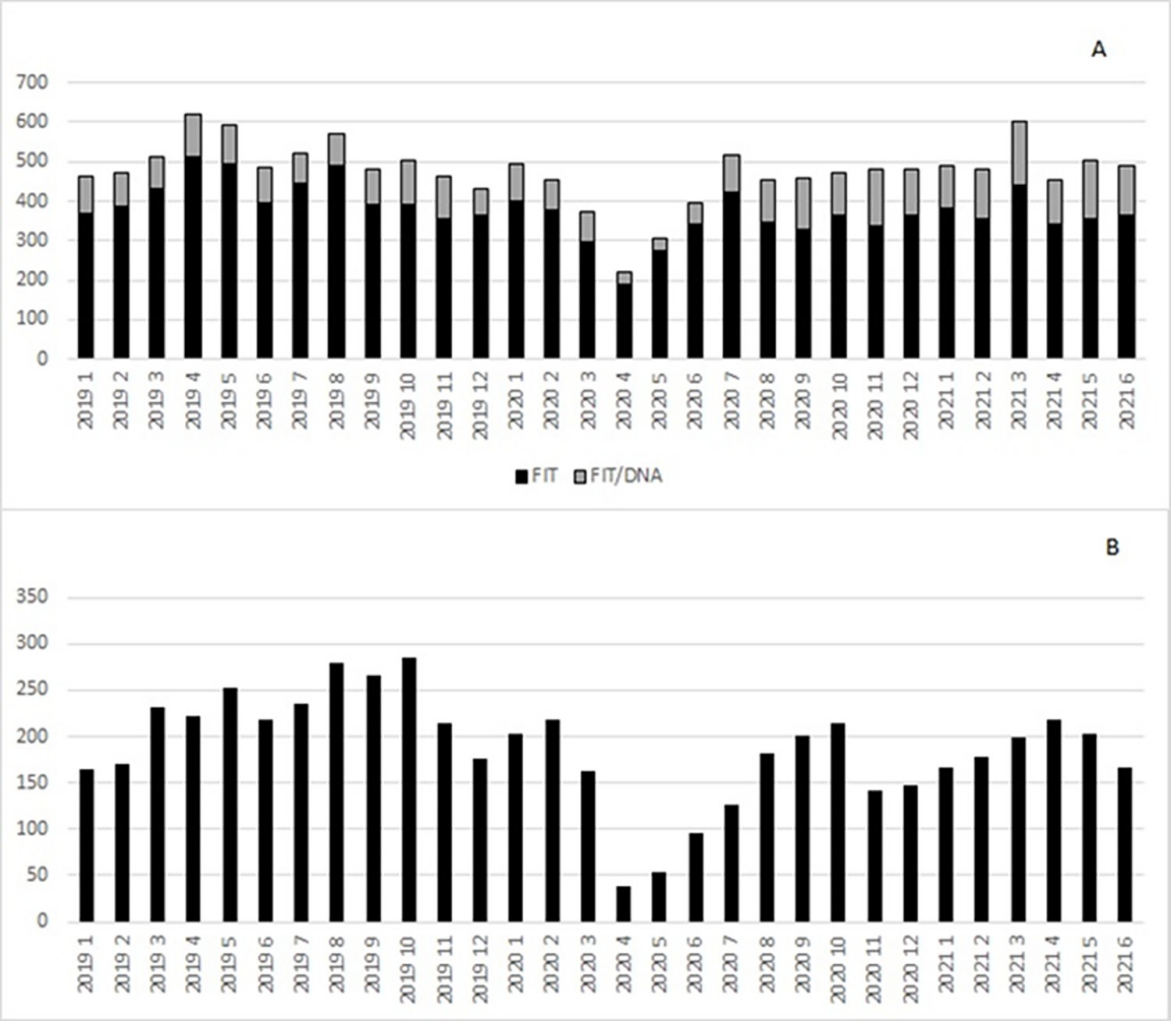
Noninvasive CRC screening tests completed per month. **A**. Private health system (FIT and FIT-DNA) and **B**. Public health system (FIT only).

**Fig 4:**
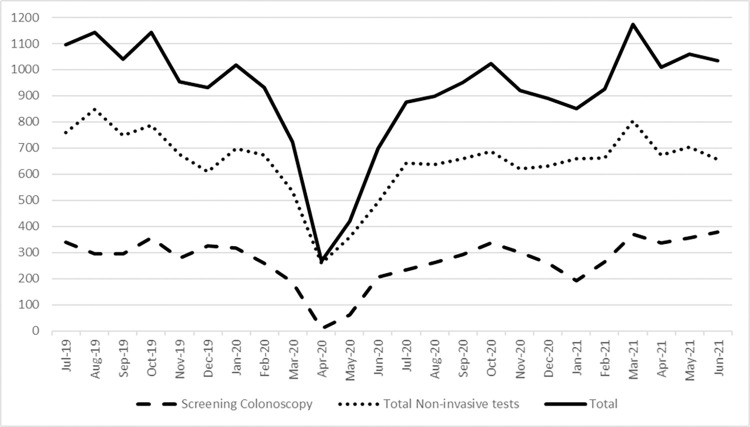
CRC screening by month (public and private health systems combined).

Comparison of completed non-invasive tests showed a decline of 21.9% (from 8,828 to 6,895) in 2020 compared with 2019. The total number of FITs declined by 24.8% in 2020 compared to 2019 (from 7,748 to 5,825), while FIT-DNA testing remained constant (from 1,080 to 1,070) (**Figs 3 and 4**).

Comparison of the number of tests per month during years 2019, 2020, and 2021 showed that there were statistically significant differences in trends over time by type of test (p<0.001). The number of monthly FITs decreased from a mean of 58.5 tests per month in 2019 to 27.2 tests per month in 2020 and 17.3 tests per month in 2021. In contrast, the number of monthly FIT-DNA tests increased from 2019 to 2021, with a mean of 91.3 monthly tests in 2019 to 112.8 tests per month in 2020 and 146.5 tests per month in 2021. Overall, there were more FIT-DNA tests on average per month than FIT, regardless of year (p<0.001). In addition, there were more noninvasive tests performed (combining FIT/FIT-DNA) in 2021 compared to 2019 and 2020 (p<0.001).

### New diagnoses of CRC and staging

A total of 360 colorectal cancers were newly diagnosed from the private hospital system during the study period, with a decline in the number of CRCs diagnosed in 2020 compared to 2019 (64 vs 134) and 2021 (64 vs 170). There were 45 (33.6%) early-stage cancers (AJCC stages I and II) diagnosed in 2019, 16 (25.0%) in 2020, and 44 (25.9%) in 2021. While 89 (66.4%) advanced stage cancers (AJCC stages III and IV) in 2019, 48 (75%) in 2020, and 126 (74.1%) in 2021 were diagnosed, there were no difference in the odds of being diagnosed with an advanced versus early-stage cancer in 2019 compared with 2020 (OR 1.62; 95%CI, 0.82–3.20; p = 0.17), or 2021 (OR 1.45; 95%CI, 0.88–2.38; p = 0.14).

### Discussion

This study quantifies and compares trends in CRC screening tests before and during the COVID-19 pandemic in two large healthcare systems located in central Indiana. Similar to several other studies [11–19], we found that there was a significant decline in colonoscopy volume during the pandemic, particularly from April to August 2020, with a 26.7% decrease in screening colonoscopies compared to 2019. However, by early fall 2020, screening colonoscopy volumes had nearly returned to pre-pandemic levels. We also observed a decline in non-invasive tests in 2020 compared to 2019, nearly all of which was due to a decline in the use of FITs, while the number of FIT-DNA tests remained stable. During the first six months of 2021, FIT monthly volumes were comparable to (January through March) or lower than (April through June) in 2019, but increased compared to 2020, while FIT-DNA tests continued to increase in 2021, which may be due to increasing use of the test in general [20].

Adherence to screening for colorectal cancer had already decreased nationally by 11% between 2018 and 2020 due to several socio-economic and demographic factors including age, gender, race, and insurance coverage [21–23]. With the onset of the COVID-19 pandemic, there was a further decline in screening colonoscopy, adding a new barrier to completion of CRC screening [24]. Although screening colonoscopy remains the gold standard test for CRC diagnosis, utilization of non-invasive tests for CRC screening, such as FIT, has been used for more than two decades by large healthcare systems in the U.S. (such as Kaiser-Permanente and Veterans Affairs) and in many European and east Asian countries [25]. Several studies have shown significant delays in CRC screening and increases in the diagnosis of advanced stage cancer [26], with predictions of increased CRC morbidity and mortality due to the COVID-19 pandemic [10, 21, 23, 26–29]. Some studies speculated that noninvasive screening tests would be utilized to a greater extent during COVID-19 pandemic [28]. Many of these the studies did not consider the trend of all CRC screening tests (i.e., invasive and non-invasive) simultaneously. Further, most studies have focused on the trend of screening colonoscopies with less consideration of whether colonoscopy for other indications (i.e., diagnostic, therapeutic, surveillance) were affected by the pandemic.

Comparing the findings of this study to studies conducted in other settings, the results are consistent with the observed decline in CRC screening and colonoscopy volumes during the pandemic [11, 18]. Other studies have reported decreases in CRC detection and colonoscopy volumes during the early stages of the pandemic. Our study adds to the existing literature by providing data beyond 2020 and by including analysis of non-invasive tests. The findings suggest that while there was a temporary disruption in CRC screening early during the pandemic, screening rebounded before the end of 2020.

A selected survey of the most recently published literature complements findings of earlier studies. A systematic review of 43 studies showed that the pandemic was associated with a 12% decrease in CRC diagnosis and treatment compared to 2019, with delays in diagnosis from 5.4% to 26% [18]. When comparing patient characteristics in 426 CRC patients diagnosed prior to the pandemic and 270 patients diagnosed during the pandemic, no difference was found in disease stage distribution during the first year of the pandemic [30]. A median delay of 3 months from positive FIT to diagnostic colonoscopy in the Irish National Bowel Screening Program had no adverse impact on clinical or histopathological outcomes [31]. Finally, a population-based, cross-sectional study with time-series analysis of diagnostic tests for several cancers showed persistent, although lessening, declines in screening and semiurgent colonoscopies but no decline in urgent or emergent colonoscopies through August of 2022 [32]. Longer term studies of CRC incidence and stage distribution from just prior to the pandemic to beyond current time are needed to understand whether and by how much the pandemic affected CRC screening and diagnosis.

Our study also examined a 2.5-year trend in newly diagnosed CRCs and their stage distribution from January 2019 to November 2021. There was a decline in the number of CRCs diagnosed in 2020 compared to 2019 and a subsequent increase in 2021. These findings contribute to the understanding of CRC trends during the study period, particularly in the context of the COVID-19 pandemic. The decline in the number of CRC cases diagnosed in 2020 compared to 2019 likely reflects the impact of the pandemic on healthcare utilization and access to screening services. The subsequent increase in 2021 suggests a potential recovery or catch-up effect in cancer diagnoses. The total number of CRC cases in 2021 was higher than in 2019, indicating a potential backlog of undiagnosed cases from the previous year. The stability in the odds ratio of advanced versus early-stage cancer diagnosis between 2019 and subsequent years suggests that the pandemic did not significantly impact the stage distribution of newly diagnosed CRC cases, although the relatively wide confidence limits reflect the small sample size. Further, the finding is somewhat unexpected, as previous studies have reported delays in cancer screenings and concerns about advanced-stage cancer diagnoses during the pandemic [13, 14, 33–35]. It is possible that the private hospital system in this study implemented strategies to maintain access to timely diagnostic services, which may have mitigated the potential effects of the pandemic on stage distribution.

The findings of our study have clinical and public health implications. The significant decline in both colonoscopy and non-invasive testing during the early COVID-19 pandemic highlights the potential consequences of disruptions in preventive care services. Delayed or missed CRC screening can lead to delayed diagnoses, resulting in more advanced-stage cancers and potentially higher mortality rates [13, 35]. The decline in CRC screening in this study was short-lived, extending from April through August of 2020. Whether and to what extent this 5-month decline in screening affects downstream CRC incidence and mortality is not known. According to a comparative microsimulation analysis that used two of the Cancer Intervention and Surveillance Modeling Network models [33], short-term delays of 3–18 months in screening and the use of FIT instead of colonoscopy were predicted to results in minor (1–2%) numbers of life years lost. While not considered in the current study, unequal recovery of screening across healthcare systems and in different settings is expected to widen disparities in CRC incidence and mortality.

One important observation of our findings involved the shift towards increased utilization of non-invasive testing with the FIT and FIT-DNA tests. Non-invasive tests can be performed at home and mailed to healthcare providers or testing centers with subsequent notification of results to the provider, eliminating the need for in-person visits and reducing the risk of exposure to infectious diseases. The increased uptake of non-invasive tests during the pandemic, as observed in our study and by others [20, 27], suggests that they may serve as an alternative to colonoscopy for certain individuals, especially during times of restricted access to healthcare facilities or during public health emergencies. Finally, we identified a nearly 25% decline in FIT use in 2020 as compared with 2019 while FIT-DNA testing remained constant in the hospital system that offered both tests. We believe this difference in trends may be due in part to overall trends in FIT-DNA use and to the convenience of ordering the test, as it is expressed mailed to the patient’s home.

Our study has several strengths. First, it provides longitudinal data over a 30-month period, allowing us to examine trends in both colonoscopy and non-invasive testing beyond the initial months of the pandemic. Second, we analyzed data from two large, integrated healthcare systems, providing a comprehensive view of screening practices in the central Indiana area. Third, the inclusion of both invasive and non-invasive testing methods allows for a more comprehensive understanding of the impact of the pandemic on CRC screening [22]. This study is among a few that consider the effect of the COVID-19 pandemic on invasive (i.e., colonoscopy) and non-invasive stool-based tests. Fourth, we examined colonoscopy indication beyond screening (i.e., diagnostic, surveillance, and therapeutic), showing much smaller declines in colonoscopy for diagnostic and surveillance indications.

This study also has limitations. First, the study was conducted in a specific geographical (urban) area and likely does not represent screening trends in suburban and rural regions, and may not represent trends in other urban regions. Second, although we observed an increase in non-invasive testing during the pandemic, we cannot determine whether the pandemic was mostly or solely responsible for the shift, or whether trends towards more non-invasive screening would have occurred anyway. Qualitative research is needed to understand patient preferences and the factors influencing the choice of screening tests. Third, the study did not examine the impact of the pandemic on adherence to diagnostic colonoscopies for a positive non-invasive test, potentially underestimating the decline in overall colonoscopy volume. Fourth, longer follow-up of incident CRC cases may have identified modest increases in incidence or a shift to more advanced disease stages.

This study highlights the need for strategies to mitigate the impact of future disruptions—pandemics or other—on healthcare services. Healthcare systems need to have ready access to robust telehealth services that include preventive services, including CRC screening. Public health campaigns and initiatives are crucial for raising awareness of the importance of CRC screening and the availability of non-invasive test options.

## Conclusions

This study provides data over a 30-month period examining trends in CRC screening prior to and during the first 18 months of the COVID-19 pandemic in two distinct university-affiliated healthcare systems. The decline in both colonoscopy and non-invasive testing during the first five months of the pandemic underscores the importance of addressing barriers and ensuring access to preventive care services. Moving forward, it is crucial to develop strategies that promote the use of non-invasive testing while maintaining adequate access to diagnostic colonoscopy for individuals with positive screening results. Long-term surveillance is needed to assess the potential downstream effects of the pandemic on colorectal cancer incidence, stage distribution, and mortality.

Future studies should focus on evaluating the effectiveness of remote monitoring technologies and telehealth interventions in facilitating colorectal cancer screenings. Subsequent research should explore strategies to address disparities in screening uptake, particularly among vulnerable populations that may be disproportionately affected by disruptions in healthcare services. By understanding the impact of the pandemic on CRC screening and through implementation of targeted interventions, it may be possible to reduce the burden of CRC and improve overall population health.

## Abbreviations

CRC: Colorectal cancer
FIT: fecal immunochemical test
AJCC: American Joint Committee on Cancer
